# UNMET NEEDS AMONG ADULTS AND OLDER ADULTS IN BRAZIL, ELSI-2019

**DOI:** 10.1093/geroni/igac059.1328

**Published:** 2022-12-20

**Authors:** Flavia Andrade, Xiayu Chen

**Affiliations:** University of Illinois--Urbana-Champaign, Urbana, Illinois, United States; University of Illinois at Urbana-Champaign, Urbana, Illinois, United States

## Abstract

Many older adults in Brazil struggle to receive adequate assistance in later life. To better understand this issue, this study examines the prevalence and characteristics of unmet needs of adults ages 50 and older with difficulties performing activities of daily living (ADL), instrumental activities of daily living (IADL), the caregiving patterns, and the consequences of unmet needs in Brazil. We use data from the second wave of the Brazilian Longitudinal Study of Aging (ELSI-Brazil) study conducted in 2019-2021 (N=9,949). Results indicate that the prevalence rate of ADL was 8.9% (95% CI 7.6 – 10.5), and the prevalence of IADL was 15.6% (95% CI 13.1 – 18.5). Among those with ADL difficulties, 11.5% (95% CI 8.2 – 16.0) reported having unmet needs. Among those with IADL difficulties, 8.8% (95% CI 6.1 – 12.4) reported having at least one activity with unmet needs. Unmet needs were higher among younger adults, those living alone, Black or mixed race, or the Northeast region. Most of the care (87%) was provided by family members, primarily daughters, and wives. About 20% of caregivers had to stop working or studying to provide the needed care. Those with ADL unmet needs were less likely to visit friends or family, whereas IADL unmet needs were associated with lower odds of visiting a doctor. Our study provides the first glimpse of the characteristics of those with currently unmet needs among adults in Brazil and of caregivers. Unmet needs are associated with social isolation and worse healthcare use.

